# Delivering yoga to people with hypertension in the UK: A qualitative study to explore yoga providers' knowledge, experiences, and attitudes

**DOI:** 10.1002/hsr2.1260

**Published:** 2023-05-15

**Authors:** Gamze Nalbant, Zeinab M. Hassanein, Sarah Lewis, Kaushik Chattopadhyay

**Affiliations:** ^1^ Lifespan and Population Health Academic Unit, School of Medicine University of Nottingham Nottingham UK; ^2^ The Nottingham Centre for Evidence‐Based Healthcare: A JBI Centre of Excellence Nottingham UK

**Keywords:** hypertension management, qualitative study, yoga providers, UK

## Abstract

**Background and Aims:**

Yoga has become increasingly popular in the world and the UK for improving health and well‐being. A growing body of research suggests that yoga could be used to improve the management of hypertension in addition to current management strategies. Previous cross‐sectional studies have also reported that hypertension is one of the most commonly disclosed health conditions in yoga sessions in the United Kingdom. Therefore, semi‐structured qualitative interviews were conducted with yoga providers in the United Kingdom (*n* = 19) to explore their knowledge, experiences, and attitudes toward delivering yoga to people with hypertension.

**Methods:**

Interviews were audio‐recorded, transcribed verbatim, and analyzed thematically.

**Results:**

Eight themes were identified. Yoga providers were generally aware of the health conditions of their attendees, and they had a reasonable knowledge of the causes, signs and symptoms, and management of hypertension. While most had received some information about hypertension as part of their initial yoga teaching training, this was generally felt to be limited. They mentioned the biopsychosocial benefits of yoga on hypertension but also expressed their concerns about the lack of regulation, the wide disparity in what is being delivered under the label of yoga, and the competency of some yoga providers.

**Conclusion:**

The findings suggest that yoga provision in the United Kingdom should be regulated with a better link with health service providers. A manual and training for yoga providers in the United Kingdom for managing hypertension using yoga would be helpful to address the training needs of yoga providers. However, there is a need for more robust studies before recommending the implementation of yoga in the management of hypertension in the United Kingdom.

## WHAT IS KNOWN

1. Previous systematic reviews showed that yoga may be effective in the management of hypertension.
2. Previous cross‐sectional studies have also reported that hypertension is one of the most commonly disclosed health conditions in yoga sessions in the United Kingdom.

## WHAT IS NEW

1. Yoga providers had received some information about hypertension as part of their initial yoga teaching training but this was generally felt to be limited.
2. Yoga providers expressed their concerns about the lack of regulation, the wide disparity in what is being delivered under the label of yoga, and the competency of some yoga providers.

## IMPLICATIONS

1. Yoga provision in the United Kingdom should be regulated with a better link with health service providers.
2. A manual and training for yoga providers in the United Kingdom for managing hypertension using yoga would be helpful to address the training needs of yoga providers. However, there is a need for more robust studies before recommending the implementation of yoga in the management of hypertension in the United Kingdom.

## 1 INTRODUCTION

Hypertension or high blood pressure is a global public health concern which is a major risk factor for stroke, myocardial infarction, heart failure, and chronic kidney disease^1^ and thus, one of the most important, treatable causes of premature morbidity and mortality in the world.^2^ It is highly prevalent, and around one in six people have hypertension worldwide.^3^ In the United Kingdom, hypertension is one of the most common health conditions, and around one in four people have hypertension.^4^ It is the third biggest risk factor for disease and disability in England after smoking and poor diet.^4^

A growing body of research suggests that yoga, a mind‐body practice that originated in the ancient Indian subcontinent, could be used to improve the management of hypertension in addition to current management strategies, predominantly, lifestyle modification and antihypertensive medication. Several systematic reviews have shown that yoga can reduce high blood pressure.^5^, ^6^, ^7^, ^8^ A recent systematic review and meta‐analysis of yoga interventions found that yoga can achieve a 6.49 mmHg reduction (95% confidence interval [CI]: 4.04–8.94) in systolic blood pressure and a 2.78 mmHg reduction (95% CI: 1.45–4.11) in diastolic blood pressure compared to control.^5^ Furthermore, yoga has become increasingly popular^9^ and is being used as a therapeutic approach by many attendees to improve their well‐being and manage health conditions in the world^10^, ^11^, ^12^ and the United Kingdom.^13^, ^14^ Previous cross‐sectional surveys conducted in the United Kingdom^15^, ^16^ and other countries such as the United States and Japan^11^, ^12^, ^17^ have also reported that hypertension is one of the most commonly disclosed health conditions in yoga sessions and perceived benefit of yoga for hypertension was high among the attendees.^13^, ^18^, ^19^

Yoga is widely practiced in South Asian countries such as India and Nepal and it has been integrated into the health care system.^20^, ^21^, ^22^, ^23^ For example, in India, there are initiatives to regulate higher education and practice. There are various levels of yoga‐related education in India, including a bachelor's degree, which is a recognized medical course^24^ and postgraduate taught and research degrees.^25^ There are also some regulations in place to guarantee the quality of yoga sessions.^22^, ^23^, ^26^ The Indian government initiated the Scheme for Voluntary Certification of Yoga Professionals, which is monitored by the Ministry of Ayush^27^, ^28^ and is creating a regulatory body for higher education and practice.^26^, ^29^

In the United Kingdom, yoga is delivered by a combination of yoga teachers and yoga therapists, that is, yoga teachers with additional training and experience in the therapeutic adaptation and application of yoga to those with health issues.^30^ Since yoga is largely unregulated in the United Kingdom, yoga teachers may be heterogeneous in their training and skills. Despite yoga's potential benefits for managing hypertension and its popularity among people with hypertension, little is known about the knowledge, experiences, or attitudes of these UK yoga providers in relation to managing hypertension in their sessions. In the process of generating evidence, yoga providers' inputs would be relevant and useful for decision‐makers as yoga providers' views as service providers would help to understand the potential issues in using yoga for the management of hypertension in the United Kingdom and ways to address the challenges. Hence, this study aimed to explore what yoga providers in the United Kingdom know about hypertension, its management, and the potential benefits of yoga for people with hypertension, what are their experiences of and attitudes to delivering yoga to people with hypertension, and what further training may be needed.

## 2 MATERIALS AND METHODS

### 2.1 Study design, location, period, and reporting

This phenomenological qualitative study was conducted in the United Kingdom from March to September 2021. Reporting of this study was guided by the Consolidated Criteria for Reporting Qualitative Research (COREQ‐32) guideline.^31^

### 2.2 Study participants and recruitment

All the 404 yoga providers in the United Kingdom who participated in a previous online cross‐sectional survey were eligible to take part in this qualitative study.^15^ Yoga providers were approached through professional yoga associations in the United Kingdom. Five yoga associations were approached to circulate the survey among their members, and four agreed, namely, the Association for Iyengar Yoga in the United Kingdom and Republic of Ireland, the British Wheel of Yoga (BWY), Complementary and Natural Health Council (CNHC), and Independent Yoga Network (IYN). Yoga providers were provided with information about this qualitative study and contact details of the lead researcher (GN) at the end of the online survey. Twenty‐two yoga providers were interested and contacted the lead researcher but three of them did not respond to the follow‐up emails. A link to the participant information sheet and consent form, created using Microsoft Forms was emailed to them. They were given at least 24 h to decide to participate in this qualitative study and the date for the interview was arranged. Further verbal consent was taken at the beginning of the interview. Participants were informed that transcripts would be anonymized and treated confidentially and that they were free to withdraw at any point during the interview if they so wished.

### 2.3 Interview guide

A semi‐structured interview guide was developed by the researchers through a series of discussions, based on the aim of the qualitative study. The interview guide was reviewed by a senior qualitative researcher (MB) and revized in line with her recommendations. It was also pretested among two yoga providers (yoga teachers) but no changes were required following piloting. The data from pilot interviews were not included in the study. The interview guide included the following three sections: (i) what do yoga providers know about hypertension, its management, and the potential benefits of yoga for people with hypertension; (ii) what are their experiences in delivering yoga to people with hypertension; and (iii) what are their attitudes to delivering yoga to people with hypertension.

### 2.4 Sample size

We intended to interview 20 yoga providers, aiming to achieve saturation of themes (i.e., the point where no new or relevant themes were discovered in the analysis). Saturation is often described as the point in data collection and analysis when new incoming data produces little or no new information to address the research question.^32^ However, the saturation of themes was achieved at the 15th interview, and we continued to interview a few more yoga providers to ensure that any important theme was not missed.

### 2.5 Interview procedures and transcription

All the interviews were conducted by the lead researcher, trained in qualitative research methods, in English using the interview guide. Interviews were conducted through video conferencing or phone calls due to the COVID‐19 pandemic. With the consent of the participants, the interviews were digitally recorded. Field notes were also taken to complement interviews in terms of nonverbal cues such as gestures and the tone and pitch of voice that may not have been adequately captured through the audio recording. Five recordings were transcribed verbatim by the lead researcher (GN) to familiarize herself with the data, and the remaining 14 recordings were transcribed by an external specialist company after signing the nondisclosure agreement. The quality of the transcripts was ensured by the lead researcher listening to the recordings and constantly comparing them to transcripts to rule out the possibility of missing data. Any potential identifiers were removed by the lead researcher and each transcript was labeled with a number.

### 2.6 Data analysis

Transcripts were imported into NVIVO 12 (QSR International, Australia) software to enable data storage and organization for analysis.^33^ Data were analyzed thematically using an inductive approach so that the researchers could determine the important concepts from the participants' perspectives.^34^ As an initial step to aid familiarization with the data, the first five interviews were read several times by two independent researchers (GN and ZMH) and then the transcripts were coded line by line. The preliminary codes were discussed between them and if any discrepancies were found, these were discussed with senior study authors (SL and KC). Then, codes were organized into overarching categories, which formed themes. Codes and themes were subsequently discussed among all the researchers resulting in an initial thematic codebook. The preliminary themes were then applied and refined following analysis of the remaining transcripts, which were independently analyzed by the same two researchers (GN and ZMH). All the researchers reviewed themes to ensure that they were distinct and not overlapping. The themes were further considered in relation to the whole data set to ensure they accurately reflected the data set. The analysis was iterative rather than a linear process and all these codes were refined during analysis as the data emerged. Each theme was described with supporting quotes that were chosen based on the best illustration of the notion of the theme. Quotes mentioned in the results section are verbatim unless indicated by an ellipsis (…) to signal that small segments of text have been removed for clarity. Participants did not provide feedback on the findings.

### 2.7 Reflexivity

Interviewees had no prior relationship with the interviewer. The lead researcher is a female and a PhD student doing a program of work around yoga for hypertension. During data collection, though she attended yoga classes in the United Kingdom, she did not have prior knowledge and experience of yoga teacher training in the United Kingdom. This enabled her to approach the participants with an open attitude even though at some points, she felt like an outsider to the yoga providers group in the United Kingdom. Therefore, before attempting to analyze the collected data, she took an effort to grasp the overall scenario of training of yoga teachers in the United Kingdom and she completed yoga teacher training in the United Kingdom and is now a qualified yoga teacher. This helped her to understand the data better, for example, made her familiar with the jargon used by the participants. During the interpretation of data, potential biases were avoided by involving independent senior researchers (SL and KC).

## 3 RESULTS

### 3.1 Participants

Interviews lasted 49 minutes on average. Nineteen yoga providers were interviewed who were generally based in England but some were also delivering online yoga sessions to people in other parts of the United Kingdom. They were delivering yoga sessions in public, private, and public/private settings. The majority of them mentioned that they did not link their practice to a particular school or style of yoga but generally called their style Hatha yoga and some of them mentioned specific branches of Hatha yoga. Yoga providers said that attendees were of mixed abilities, mixed sexes, and mainly adults with a broad range of health issues (Table 1).

**Table 1:** Sociodemographic characteristics of the interviewees.

Characteristics	*N*
Yoga providers	
Yoga teacher	18
Yoga therapist	1
Sex	
Male	1
Female	18
Age, in years (range)	25–73
Teaching experience, in years (range)	1.5–30
Settings^a^	
In private settings (e.g., yoga studios, gyms, health clubs, and attendees' homes)	14
In public settings (e.g., leisure centres, community halls, church halls, GP surgeries, and hospital wards)	10
In public/private settings (e.g., online, outdoors)	8
Style of yoga^a^	
Gentle yoga	1
Hatha yoga	13
Iyengar yoga	1
Jivamukti	1
Sivananda	2
Satyananda	1
Therapeutic yoga	1
Viniyoga	1

^a^ Participants described more than one style and setting.

### 3.2 Thematic analysis

Eight themes were identified.

#### 3.2.1 Theme 1: Awareness of health conditions

Yoga providers generally asked their attendees if they had any injury or health issues at the beginning of each class, or the stage of an individual joining a new class. Some asked for a verbal disclosure of any health issues, whereas others asked them to fill in a written health form. However, some yoga providers added that it was not always possible to ask attendees if they had any health issues, for example in gyms where sessions are drop‐in or when collecting health information in written form was not allowed by the center. In addition, the difficulty of asking attendees to disclose their issues in an online class was mentioned as the attendees were only able to see the yoga provider or were muted throughout the session.

*They have to fill out a form so that I'm fully aware of what*—*well everything from their challenges you know, what issues they've got, what they want to get out of yoga and what their aims are*. (P18)

*Sometimes, when you're teaching in a space that's open, people just turn up and that can be a bit challenging*. (P14)

A variety of health issues were disclosed by attendees to the yoga providers including musculoskeletal issues, mental health issues such as stress, anxiety and depression, hypertension, and cancer. Stress and hypertension were among the most commonly disclosed conditions along with musculoskeletal issues such as joint or back pain. Most yoga providers reported that at least some of their attendees disclosed hypertension to them and the percentage who did so varied from 1% to almost 100%. It was highlighted that awareness of health conditions was dependent on disclosure, and attendees might not have disclosed hypertension either because they were not aware of it themselves or because they did not perceive it as relevant to mention to the provider.

*I've had high blood pressure, low blood pressure, stress, anxiety, depression… A lot of joint problems knees, hips, wrists, and things*. (P1)

*The ones declared, it's quite low…It's not as high as I thought it would be. That's why I think either they're not saying because they think, oh it's not important, or some people don't like to admit particular conditions that they've got and others, they just don't know they've got it*. (P5)

#### 3.2.2 Theme 2: Variability in knowledge or understanding of hypertension

Yoga providers generally had some concept of the causes, symptoms, and management of hypertension though a few of them acknowledged they were not very knowledgeable about these. They mostly perceived hypertension to be associated with stress, but some listed that age, ethnicity, genetic factors, lifestyle (including unhealthy diet, sedentary lifestyle, smoking, and alcohol consumption), some health conditions and medicines, and thickening of arteries can be causes of hypertension. Some mentioned that being aware of the condition is the first step in its management. Many yoga providers perceived that having a healthier lifestyle including exercise, moderation in diet, smoking and alcohol consumption, and medication would help in its management. In addition, they noted that yoga and any activity that helps reduce stress helps to manage hypertension.

*My understanding is that stress is a key factor in increasing blood pressure*. (P11)

*Breathing, meditation, changing your diet, changing your lifestyle, being in nature… allows you to manage it*. (P15)

Many yoga providers learned about hypertension as part of their general yoga training. However, it was not covered in enough detail but rather often involved a list of contraindications. This was because the training encompassed a vast amount of information covered in a short timeframe, of which, managing hypertension was only one aspect. Some of the yoga providers knew about hypertension from a course undertaken after their general training, or through personal life experiences or personal interests and a few of them were health service providers so they already had background knowledge. Yoga providers were not aware of any specific training or guidance on the use of yoga for hypertension. In addition, although they suggested that yoga could help manage hypertension, many of them were not aware of any evidence of the effectiveness of yoga on hypertension. Most suspected there must be relevant evidence available even though they had not searched for it. A few of them had explored the published literature and were aware that the current evidence is limited. One yoga provider who had some knowledge of the available evidence questioned the quality of available research studies. Some yoga providers suggested that more research into the benefits of yoga for hypertension was probably needed.

*I did read a study. The thing is a lot of the studies that you read are very small and a lot of them are very contradictory. So you can read one that says yes it's brilliant for this and another one says it is rubbish*. (P3)

*I actually believe yoga is beneficial, and I also believe that we need more scientific research to show that… Because people just go, “Oh yes, well it's all in the head”. Science likes to have factual evidence‐based research, so that's why I wanted to participate*. (P6)

#### 3.2.3 Theme 3: Variability in yoga and standards of yoga available

When asked about the style of yoga practiced, yoga providers generally referred to Hatha yoga, but there are various styles created out of this type of yoga. Many of them mentioned that yoga has rich and diverse styles but surmised that not all these practices would be suitable for those with hypertension. Therefore, finding the right class for people with hypertension was deemed important.

*Yoga is such a massive title; a yoga class could mean so many different things and so I think it's really important that people check with the teacher and make sure that what they're doing is yoga that's suitable for hypertension*. (P7)

Most yoga providers expressed their concerns that yoga is not regulated in the United Kingdom and there are no set requirements to be a yoga provider resulting in the delivery of yoga classes by those with no proper training. There was concern that guidance by a trained yoga provider was important to get benefits from yoga and avoid any potential harm that could be caused to people with hypertension. Yoga providers spoke of the important characteristics of such a yoga provider, not only having years of teaching experience but also having experience in teaching to a particular population and being open to learning and engaging in ways of helping the variety of health issues they came across. It was suggested that yoga provision should be regulated and accredited for the safety of the attendees, the credibility of the yoga providers, and the assurance of health service providers (e.g., clinicians and nurses).

*Yoga isn't registered, yoga teachers aren't registered so you can be somebody that's gone on holiday to Spain for two weeks and done a yoga teaching course for two weeks and you can come back, and you can be a yoga teacher*. (P3)

*If you are under the right guidance, yoga will not be harmful but if you are under the wrong guidance, it may be harmful in that way*. (P15)

#### 3.2.4 Theme 4: Perceived benefits of yoga for people with hypertension

Yoga providers shared some of their experiences and spoke of positive feedback received from their attendees in relation to the effects of yoga on their blood pressure and some of them even exemplified that their attendees reduced their medication during their yoga journey. Many yoga providers were aware that yoga could help manage hypertension through its physiological, physical, and mental benefits. Some yoga providers had more detailed knowledge of some of the mechanisms by which yoga can be beneficial, for example, its effects on the nervous system. Some perceived that yoga helps to improve physical strength and self‐awareness of people about their bodies and leads them to make healthier lifestyle choices. Yoga's contribution to mental health through its calming and stress‐reducing effects was especially highlighted by most yoga providers as stress was seen as one of the major contributors to hypertension. It was also added that doing something beneficial for themselves makes attendees feel better and some of them said that yoga enhances the feeling of a sense of community and acceptance, which contributes to emotional well‐being.

*There is no medical evidence but what there is, a lot of people tell me, they feel better, they use less medication, they stop using painkillers, they manage their lifestyle a lot better, having come to yoga for a period of months or years*. (P11)

*It is definitely stress reduction and also yoga can put you in a different frame of mind and that sort of self‐care perspective that can have a knock‐on effect on how you manage the rest of your life…* (P16)

Most yoga providers perceived that gentler styles of yoga where postures were held for longer such as Restorative Yoga, Yin Yoga, and Gentle Hatha Yoga rather than fast‐flow yoga styles would be more beneficial for people with hypertension. Some believed that doing any yogic practice, for example, asana, pranayama, or dhyana and relaxation practices, would help people with hypertension whereas some emphasized the importance of pranayama practices for lowering blood pressure such as breath awareness, exhalation longer than inhalation, and Sheetali pranayama. However, several yoga providers mentioned the incorporation of these three yogic practices, that is, yogic poses, breathing practices, and relaxation and meditation, in a session to get the maximum benefit.

*I think that the whole process of yoga practice could potentially be helpful, certainly in terms of encouraging people to become aware of their breath and to use their breath, particularly their exhalation, to perhaps try and stimulate the parasympathetic nervous system to calm the whole system*. (P19)

*The combination of everything together, you know the movement, the breathing, the focused concentration…so it's really kind of bringing everything together to a point of focus to hopefully calm things down…* (P2)

#### 3.2.5 Theme 5: Perceived harms from yoga or contraindications

Yoga providers suggested there were some yoga styles and practices that needed to be modified or avoided totally for people with hypertension. Yoga styles practiced in a hot environment and with dynamic fast flow practices such as Hot/Bikram, Ashtanga, Vinyasa, and Power yoga were not advised by most yoga providers for an attendee with hypertension. In terms of yogic practices, it was related that any asana taking the head lower than heart levels such as headstand, handstand, shoulder stand, and deep back arches are contraindicated, and holding arms above the head for long periods might not be advisable. For example, some mentioned that they offer a half‐forward bend instead of a forward bend where the head is kept below the heart level. Pranayamas including breath retention and forceful breathing such as Bhastrika and Kapalabhati were also considered risky to be practiced by people with hypertension.

*Hypertensive people need to be very careful which type of yoga they do. So there has to be a distinction between fitness yoga and therapeutic yoga*. (P17)

*I would advise against going to hot yoga, fast‐flowing Ashtanga, or Vinyasa yoga where you link all the poses together and work quite quickly. I would advocate against strong working back‐arching poses where you're working very actively to open up the front chest, like back arches*. (P8)

Yoga providers perceived that without the necessary modifications, there was a chance of increasing blood pressure. It was suggested that yogic practices should be introduced working from basic to advanced levels and attendees should be observed for any visual clue of struggle to adapt practices to the individuals' needs. Though necessary modifications were generally provided, they tried to teach their attendees to be aware of their bodies and recommended knowing their limitations and adapting accordingly. Some mentioned that attendees can be very competitive and may not pay attention to the modifications offered to them. Most yoga providers had not experienced any adverse events with people with hypertension, but some mentioned very mild issues such as dizziness and light‐headedness experienced when the attendees did not pay attention to the modifications offered.

*You need to make adjustments for people, which is why it's really important to watch and adjust your students and observe them during classes and if they're struggling with something*. (P7)

*People sometimes have a competition with themselves, they go to a class, and they think that everyone should be doing the same thing, the same way where it's not like that but they don't want to be the first person to come out of a posture*. (P12)

#### 3.2.6 Theme 6: Views on how yoga can be best delivered to people with hypertension

Yoga providers varied in their opinions on whether people with hypertension need separate classes. Some yoga providers expressed concerns that if the number of people with hypertension attending a class increases markedly as a result of referrals by health service providers, they would plan to set up a special class for them or they would have one‐to‐one sessions as they might be otherwise ignoring other attendees' needs. However, many yoga providers suggested that people with hypertension could be managed in a group class as all attendees in a class had some kind of issues and hypertensives were not any different from others. They also mentioned that setting up a special class for them might make some attendees feel isolated from others and might give them false hope that their hypertension could be cured by these yoga classes.

*…if the adaptation actually made the whole class better for everybody, then, that would be great. But if those adaptations made that I was taking out things that other people value in class, then, that would be my challenge*. (P4)

*Equally, that then puts them into some kind of awful, “we're the hypertensive group” and maybe yoga might help them not to be hypertensive anymore, so I would prefer to absorb them into a mainstream class. Why not?* (P19)

#### 3.2.7 Theme 7: Gaps in yoga training in relation to managing hypertension and ways to address these gaps

The information provided during the training of yoga teachers was adequate to deliver a generic yoga session, but yoga providers suggested that further training was needed to build their capacity for managing hypertension.

*When you start a teacher training, I think there is such a vast amount of knowledge to cover; you can only touch on each of the individual topics that you need to have a grasp of. I would not put detailed training about using yoga for hypertension in a general training course*. (P13)

*All it said was you don't do shoulder stand and headstand if you've got high blood pressure. I think that's more or less all it covered really; just certain postures aren't appropriate for certain medical conditions*. (P5)

Yoga providers were generally happy to include people with hypertension in their sessions as they already modified their practices according to their attendees' needs. Some spoke of self‐educating themselves to provide safer practices by searching online but others also complained about the contradiction of information available online. Many also expressed uncertainty about whether adaptations and contraindications were relevant when people with hypertension are medicated. They, therefore, suggested that further formal training such as a day‐long workshop or a continual professional development course would be helpful to refresh and deepen their knowledge, especially if they were to set up a special class for hypertensive people. The training could cover evidence‐based up‐to‐date information on how yoga providers could help people with hypertension, the rationale behind certain modifications, and the medical side of hypertension, for example, if there were differences between medicated and nonmedicated attendees in terms of what practices they could do.

*…having a module for that would be, or even just a short course… just to teach that would be quite helpful I think, to make sure we're all doing the right thing and what we're doing is backed up because everybody has their own ideas I think at the moment*. (P5)

*What would be good to know is why exactly shouldn't a hypertensive person do inversion … if I understood more about that the why then, I could apply that knowledge to other things as well*. (P4)

*What I don't know is to what extent medication balances things out for people with hypertension. I was taught that if somebody is on medication for hypertension then often, they're fine to do a lot of yoga practices…* (P18)

#### 3.2.8 Theme 8: The need for a better link with health service providers

Yoga providers felt strongly that yoga should be encouraged more widely by the government and NHS, and in particular, health service providers should have a role in encouraging their patients to try yoga, as health service providers tend to be more highly trusted. A couple of them mentioned that there were some local initiatives where GPs referred their patients to yoga classes. However, some yoga providers talked of the health system not yet recognizing the potential of yoga for managing hypertension and other conditions and they expressed that NHS is very slow with coming on board and recognizing yoga as a health management and wellbeing tool. It was exemplified by a yoga provider who was aware of a major research project on yoga for healthy lower backs going on for more than 10 years but still not recognized by the NHS.

*I think it would be their health care professionals because also that would hopefully give it credibility that it could be suggested*. (P4)

*It still hasn't quite got into the mainstream GP surgeries, hospitals, and NHS. They're still not quite recognising it. It's getting there, but it's taking such a long time, partly because the NHS needs to be convinced of these alternative therapies, they might call them, of being effective…* (P5)

Some yoga providers acknowledged the difficulty for health service providers to recommend the right yoga classes to their patients among the plethora of offers available and that there was a need for more evidence‐based research to convince the NHS of the benefits of yoga on hypertension management. Many yoga providers, therefore, suggested that if partnerships with health service providers were established, it would help in terms of offering the right yoga practices to people with hypertension and would save time and money for the NHS as yoga would promote self‐management of hypertension and hypertension‐related issues. Some yoga providers talked of a more collaborative approach that might be used, for example, the idea of social prescribing, that is, prescribing yoga as a way of managing a medical problem.

*From a community perspective, obviously GPs and specialist nurses could certainly start prescribing it. It would be great if they even held a yoga clinic at their GP surgery, or directed them to. I think there might be some link in terms of, I don't know if it quite comes under social prescribing…* (P16)

## 4 DISCUSSION

This qualitative study provided unique insights into the knowledge, experiences, and attitudes of yoga providers in delivering yoga to people with hypertension in the United Kingdom. We could not find any study exploring the knowledge of yoga providers on hypertension and their experiences and attitudes to delivering sessions to people with hypertension in the world including the Indian subcontinent where yoga originated and is widespread. Many yoga providers were aware of the health conditions of their attendees though this was dependent on disclosure, and they had a reasonable knowledge of the causes, signs and symptoms, and management of hypertension. While most had received some information about hypertension as part of their initial yoga teacher training, this was generally felt to be limited. Many of them were trying to meet the needs of those with hypertension for example by making adaptations within the yoga sessions but they generally lacked confidence in what they were advising for people with hypertension. Interestingly, though hypertension is a prevalent health condition and people with hypertension are frequently attending yoga sessions in the United Kingdom,^4^, ^15^, ^16^ we could not find any yoga manuals or training on hypertension management using yoga. Yoga providers were also unaware of such a manual or training. Yoga providers, however, mentioned the availability of training and sessions for cancer, back pain, and pregnancy in the United Kingdom.

There are many studies suggesting the effectiveness of yoga interventions for managing hypertension^5^, ^6^, ^7^, ^8^ but yoga providers mainly were not aware of such evidence which may suggest that they mostly do not incorporate evidence‐based findings into their sessions. This is probably because it is difficult for them to locate and utilize the data on the most effective and safe yogic practices. They also tend to lack the knowledge and skills how to critically appraise research articles, therefore, they can struggle to make sense of the results from studies, which are at times contradictory in their findings.^35^, ^36^ Thus, a manual and a training program based on the manual for yoga providers in the United Kingdom for managing hypertension would be helpful to address their training needs. However, a previous systematic review found some evidence of the effectiveness of yoga in managing hypertension, but the quality of the included studies was poor.^5^ This review also synthesized the heterogenous content, structure, and delivery characteristics of effective yoga interventions used for hypertension management.^5^ Therefore, the subsequent step would be to attain a consensus over the findings of this review to develop the intervention (i.e., intervention materials like a manual for yoga providers) and conduct a robustly designed randomized controlled trial (RCT). However, even if a manual is developed for hypertension management using yoga, initially, it may not be feasible to expect all yoga providers to adhere to it. Nevertheless, some yoga providers have searched for evidence on what practices would be helpful or contraindicated for people with hypertension but recognized that there is currently a lack of evidence in this field. This implies that some of them comprehend the concept of evidence‐based practices and may still be able to engage in evidence‐based practices to some extent.

Yoga providers highlighted the biopsychosocial benefits of yoga for hypertension, which include physical, psychological, and social benefits. For example, generally, yoga is a non‐strenuous activity practiced in a calm environment and a class with a group of people.^37^, ^38^, ^39^ These aspects of yoga were perceived by yoga providers to be making it an activity that increases mobility and physical function as well as improving the mental well‐being of attendees and providing them with a sense of community. This is in accord with earlier studies where participants reported increased social connection and more positive relations with others.^19^, ^40^, ^41^, ^42^ In addition, a systematic review and meta‐analysis showed that yoga is a reasonably safe and well‐tolerated practice with limited adverse events when practiced appropriately.^43^ As well as yoga being a potential way of managing hypertension, it may cause concern due to its potential to cause harm. For example, there are more than 20 yoga styles, and specific styles, such as Hot/Bikram yoga, may not be suitable for people with hypertension.^44^, ^45^ As suggested by yoga providers in this study, even if the right style is chosen, if yoga is not practiced correctly and under proper guidance, it may lead to adverse events. Similarly, a survey assessing yoga‐related injuries found that the most common causes were excessive effort, inadequate training of yoga providers, and improper or inadequate instruction given by yoga providers.^46^

Along with the increasing popularity of yoga and the number of yoga attendees, the number of yoga providers has also increased.^14^ As there are no nationally set standards for yoga provision in the United Kingdom,^15^ people with no adequate training can also use the title “yoga teacher.” Though yoga providers showed a willingness to teach yoga to people with hypertension, concerns have been raised about the lack of regulation and the competency of some yoga providers.^47^ The lack of regulation may also cause hesitation by health service providers in recommending yoga to their patients. Therefore, to maintain the quality of yoga sessions and to ensure the credibility of yoga providers and the safety of yoga attendees, yoga should be regulated by a formal organization or association for example yoga associations. The lack of regulation is an issue that must be addressed for people with hypertension or other health conditions as well as for people without any health condition (i.e., as part of a healthy lifestyle to prevent diseases). Yoga is also not regulated in the United States and some other European countries such as Germany,^48^, ^49^ however, it appears that yoga has been incorporated into patient care in these countries.^48^, ^50^ For example, a study in the United States reported that 25.6% of physicians recommended yoga to their patients as a complementary health approach^50^ and another study reported that at least 14 million people in the United States started practicing yoga because a physician recommended it for their health condition.^51^ However, very little is known about the attitudes of health service providers in the referral of patients to yoga classes in the United Kingdom. A study in the United Kingdom reported a low rate of patient referral by GPs, and 14% (of 524) referred their patients to a private yoga class in 2001.^52^ Collaboration between yoga providers and health service providers in the United Kingdom was recommended by yoga providers to overcome hesitation in recommending yoga to people with hypertension among health service providers and to help people with hypertension to access a yoga session that is suitable to their needs. Yoga might be included within the wider context of what is known as social prescribing, where individuals can be linked from health providers to holistic health resources in the community aimed at improving health and well‐being. Indeed, a pilot project (Yoga4Health) was conducted in London in 2017 with the aim of social prescription of yoga for various health issues including high blood pressure in the United Kingdom.^53^ A before and after, mixed method study was used to evaluate the project and results showed a reduction in perceived stress and anxiety and improvements in well‐being and social connectedness. Improvement in blood pressure was also reported. In addition, this project was perceived as acceptable to both yoga attendees and stakeholders (such as yoga providers). The Yoga4Health project has also been expanded to other cities in the United Kingdom suggesting that it will have wider acceptability. However, before recommending the inclusion of yoga for hypertension management within social prescription in the United Kingdom, there is a need for more robust studies, for example, high‐quality RCTs to understand the effectiveness, cost‐effectiveness, and affordability of yoga in the management of hypertension, and its acceptability to people with hypertension and health service providers as well as regulating yoga practices in the United Kingdom.

### 4.1 Strengths and weaknesses

To the best of our knowledge, this was the first study that explored the knowledge, experiences, and attitudes of yoga providers in delivering yoga to people with hypertension. The interviews were conducted by the lead researcher (GN) with whom the study participants had no prior acquaintance. In addition, to ensure the dependability and credibility of the research findings, other researchers (KC, SL, ZMH) who had not conducted the interviews were involved in analytic decisions relating to data interpretation. The lead researcher (GN) also reflected on her previous experiences and their effects on decisions related to different phases of data collection and analysis. The selection and recruitment relied on voluntarism; therefore, yoga providers who were already delivering yoga sessions to people with hypertension may have shown interest in this study. Therefore, their views may not be transferrable to yoga providers across the United Kingdom or other settings. Only one yoga therapist was interviewed which provided some insights from this group of yoga providers, but the study predominantly reflected the views of yoga teachers in the United Kingdom. However, the vast majority of yoga is delivered by yoga teachers,^15^ and hence if yoga is to be more widely promoted as part of a healthy lifestyle, it is likely to be yoga teachers who need to be trained to deliver what is required.

### 4.2 Conclusion

Yoga providers showed a willingness to teach yoga to people with hypertension, however, the wide disparity in what is being delivered under the label of yoga and the competency of some yoga providers was a concern to them. Therefore, lack of regulation is an issue that needs to be addressed for people with hypertension or other health conditions as well as for people without any health condition. A manual and training for yoga providers in the United Kingdom for managing hypertension using yoga would be helpful to address the training needs of yoga providers. In addition, a better link between health service providers and yoga providers could be established to overcome the hesitation of health service providers in recommending yoga to people with hypertension and to direct people with hypertension to a yoga session that is suitable to their needs. However, there is a need for more robust evidence before the implementation and regulation of yoga can be recommended across the UK for the management of hypertension.

## AUTHOR CONTRIBUTIONS

**Gamze Nalbant**: Conceptualization; formal analysis; investigation; project administration; writing—original draft; writing—review and editing. **Zeinab M. Hassanein**: Formal analysis; writing—review and editing. **Sarah Lewis**: Formal analysis; writing—review and editing. **Kaushik Chattopadhyay**: Formal analysis; writing—review and editing. All authors have read and approved the final version of the manuscript. Gamze Nalbant had full access to all of the data in the study and takes complete responsibility for the integrity of the data and the accuracy of the data analysis.

## CONFLICT OF INTEREST STATEMENT

The authors declare no conflict of interest.

## ETHICS STATEMENT

The study was conducted in accordance with the Declaration of Helsinki and approved by the University of Nottingham's Faculty of Medicine and Health Sciences Research Ethics Committee (Ref No. 467‐2001). Informed consent was obtained from all subjects involved in the study.

## TRANSPARENCY STATEMENT

The lead author Gamze Nalbant affirms that this manuscript is an honest, accurate, and transparent account of the study being reported; that no important aspects of the study have been omitted; and that any discrepancies from the study as planned (and, if relevant, registered) have been explained.

## Data Availability

A deidentified data set will be available upon request unless there are legal or ethical reasons for not doing so.

## References

[1] National Institute for Care and Excellence . Hypertension in Adults: Diagnosis and Management; 2019. Accessed October 20, 2019. https://www.nice.org.uk/guidance/ng136/chapter/Recommendations#diagnosing-hypertension

[2] World Health Organization . Hypertension. 2021. Accessed January 1, 2023. https://www.who.int/news-room/fact-sheets/detail/hypertension

[3] Zhou B , Bentham J , Di Cesare M , et al. Worldwide trends in blood pressure from 1975 to 2015: a pooled analysis of 1479 population‐based measurement studies with 19.1 million participants. The Lancet. 2017;389(10064):37‐55. 10.1016/S0140-6736(16)31919-5 PMC5220163 27863813

[4] Public Health England . Health matters: combating high blood pressure [Internet]; 2017. Accessed August 1, 2019. https://www.gov.uk/government/publications/health-matters-combating-high-blood-pressure/health-matters-combating-high-blood-pressure

[5] Nalbant G , Hassanein ZM , Lewis S , Chattopadhyay K . Content, structure, and delivery characteristics of yoga interventions for managing hypertension: a systematic review and meta‐analysis of randomized controlled trials. Front Public Health. 2022;10:846231. 35419342 10.3389/fpubh.2022.846231 PMC8995771

[6] Hagins M , States R , Selfe T , Innes K . Effectiveness of yoga for hypertension: systematic review and meta‐analysis. Evid‐Based Complement Altern Med. 2013;2013:1‐13. 10.1155/2013/649836 PMC3679769 23781266

[7] Cramer H , Haller H , Lauche R , Steckhan N , Michalsen A , Dobos G . A systematic review and meta‐analysis of yoga for hypertension. Am J Hypertens. 2014;27(9):1146‐1151. 24795403 10.1093/ajh/hpu078

[8] Park S‐H , Han KS . Blood pressure response to meditation and yoga: a systematic review and meta‐analysis. J Altern Complement Med. 2017;23(9):685‐95. 28384004 10.1089/acm.2016.0234

[9] Wieland LS , Cramer H , Lauche R , Verstappen A , Parker EA , Pilkington K . Evidence on yoga for health: a bibliometric analysis of systematic reviews. Complement Ther Med. 2021;60:102746. 34091028 10.1016/j.ctim.2021.102746 PMC8350934

[10] Cramer H , Lauche R . Yoga therapy: efficacy, mechanisms and implementation. Complement Ther Med. 2018;40:236. 30219457 10.1016/j.ctim.2017.11.015

[11] Matsushita T , Oka T . A large‐scale survey of adverse events experienced in yoga classes. Biopsychosoc Med. 2015;9:9. 25844090 10.1186/s13030-015-0037-1 PMC4384376

[12] Quilty MT , Saper RB , Goldstein R , Khalsa SBS . Yoga in the real world: perceptions, motivators, barriers, and patterns of use. Glob Adv Health Med. 2013;2(1):44‐49. 10.7453/gahmj.2013.2.1.008 PMC3833584 24381824

[13] Cartwright T , Mason H , Porter A , Pilkington K . Yoga practice in the UK: a cross‐sectional survey of motivation, health benefits and behaviours. BMJ Open. 2020;10(1):e031848. 10.1136/bmjopen-2019-031848 PMC7044896 31932388

[14] Ali‐Knight J , Ensor J . Salute to the Sun: an exploration of UK yoga tourist profiles. Tour Recreat Res. 2017;42(4):484‐497.

[15] Nalbant G , Lewis S , Chattopadhyay K . Characteristics of yoga providers and their sessions and attendees in the UK: a cross‐sectional survey. Int J Environ Res Public Health. 2022;19(4):2212. 35206399 10.3390/ijerph19042212 PMC8871723

[16] Ding D , Stamatakis E . Yoga practice in England 1997‐2008: prevalence, temporal trends, and correlates of participation. BMC Res Notes. 2014;7:172. 24661723 10.1186/1756-0500-7-172 PMC3987846

[17] Ross A , Friedmann E , Bevans M , Thomas S . National survey of yoga practitioners: mental and physical health benefits. Complement Ther Med. 2013;21(4):313‐323. 23876562 10.1016/j.ctim.2013.04.001 PMC3721070

[18] Wolff M , Brorsson A , Midlöv P , Sundquist K , Strandberg EL . Yoga—a laborious way to well‐being: patients' experiences of yoga as a treatment for hypertension in primary care. Scand J Prim Health Care. 2017;35(4):360‐368. 29124990 10.1080/02813432.2017.1397318 PMC5730034

[19] Cheshire A , Richards R , Cartwright T . ‘Joining a group was inspiring’: a qualitative study of service users' experiences of yoga on social prescription. BMC Complement Med Ther. 2022;22(1):67. 35287676 10.1186/s12906-022-03514-3 PMC8922896

[20] Anjana RM , Ranjani H , Unnikrishnan R , Weber MB , Mohan V , Venkat Narayan KM . Exercise patterns and behaviour in Asian Indians: data from the baseline survey of the Diabetes Community Lifestyle Improvement Program (D‐CLIP). Diabetes Res Clin Pract. 2015;107(1):77‐84. 25458336 10.1016/j.diabres.2014.09.053

[21] Dhungana RR , Khatiwoda SR , Gurung Y , Pedišić Ž , de Courten M . Yoga for hypertensive patients: a study on barriers and facilitators of its implementation in primary care. Glob Health Action. 2021;14(1):1952753. 34323666 10.1080/16549716.2021.1952753 PMC8330799

[22] Shrivastava SR , Shrivastava PS , Ramasamy J . Mainstreaming of ayurveda, yoga, naturopathy, unani, siddha, and homeopathy with the health care delivery system in India. J Tradit Complement Med. 2015;5(2):116‐118. 26151021 10.1016/j.jtcme.2014.11.002 PMC4488105

[23] Dhungana RR , Khanal MK , Joshi S , et al. Impact of a structured yoga program on blood pressure reduction among hypertensive patients: study protocol for a pragmatic randomized multicenter trial in primary health care settings in Nepal. BMC Complement Altern Med. 2018;18:207. 29976188 10.1186/s12906-018-2275-9 PMC6034305

[24] Shobhit University . Bachelor of naturopathy & yogic sciences. Accessed February 6, 2023. https://www.shobhituniversity.ac.in/naturopathy-yoga/bnys-program.php

[25] Mohanty S . Yoga education for all 2020. Accessed February 6, 2023. https://www.researchgate.net/publication/346473375_YOGA_EDUCATION_FOR_ALL

[26] Parliament of India . One hundred fifteenth report on the national commission for Indian system of medicine bill, New Delhi; 2019. Accessed February 1, 2023. https://rajyasabha.nic.in/rsnew/Committee_site/Committee_File/ReportFile/14/121/115_2019_11_17.pdf

[27] AYUSH. Scheme for voluntary certification of yoga professionals started by M/o AYUSH to provide good quality Yoga teachers: Shri Shripad Yesso Naik 2016. Accessed February 1, 2023. https://pib.gov.in/newsite/printrelease.aspx?relid=147748.

[28] The Yoga Institute . Yoga as a professional career 2020. Accessed February 1, 2023. https://theyogainstitute.org/yoga-as-a-professional-career/

[29] National Assessment and Accreditation Council . Manual for yoga higher education institutional accreditation Bengaluru 2021. Accessed February 1, 2023. http://www.naac.gov.in/images/docs/Manuals/NEP_docs/Yoga_University_manual__proposed_by_EC_Final_Copy.pdf

[30] Complementary and Natural Healthcare Council (CNHC) . Yoga therapy [Internet]; 2019. Accessed July 30, 2019. https://www.cnhc.org.uk/yoga-therapy

[31] Tong A , Sainsbury P , Craig J . Consolidated criteria for reporting qualitative research (COREQ): a 32‐item checklist for interviews and focus groups. Int J Qual Health Care. 2007;19(6):349‐357. 17872937 10.1093/intqhc/mzm042

[32] Guest G , Namey E , Chen M . A simple method to assess and report thematic saturation in qualitative research. PLoS One. 2020;15(5):e0232076. 32369511 10.1371/journal.pone.0232076 PMC7200005

[33] QSR International . NVivo 12 qualitative data analysis software. QSR International; 2018. Accessed January 1, 2023. https://www.qsrinternational.com/nvivo/nvivo-12-tutorial-windows/00-let-s-get-started

[34] Braun V , Clarke V . Using thematic analysis in psychology. Qual Res Psychol. 2006;3(2):77‐101.

[35] Bhavanani A . A brief qualitative survey on the utilization of yoga research resources by yoga teachers. J Intercult Ethnopharmacol. 2016;5:168‐173. 27104038 10.5455/jice.20160331064758 PMC4835992

[36] Moonaz S , Jeter P , Schmalzl L . The importance of research literacy for yoga therapists. Int J Yoga Ther. 2017;27:131‐133. 10.17761/1531-2054-27.1.131 29131733

[37] Ramanathan M , Bhavanani A . Mental health and wellbeing in silver citizens through yoga. Eur J Biomed Pharm. 2017;4:288‐292.

[38] Cramer H , Ward L , Steel A , Lauche R , Dobos G , Zhang Y . Prevalence, patterns, and predictors of yoga use. Am J Prev Med. 2016;50(2):230‐235. 26497261 10.1016/j.amepre.2015.07.037

[39] Penman S , Cohen M , Stevens P , Jackson S . Yoga in Australia: results of a national survey. Int J Yoga. 2012;5(2):92‐101. 22869991 10.4103/0973-6131.98217 PMC3410203

[40] Kishida M , Mama SK , Larkey LK , Elavsky S . “Yoga resets my inner peace barometer”: a qualitative study illuminating the pathways of how yoga impacts one's relationship to oneself and to others. Complement Ther Med. 2018;40:215‐221. 30219453 10.1016/j.ctim.2017.10.002

[41] Ross A , Bevans M , Friedmann E , Williams L , Thomas S . “I am a nice person when I do yoga!!!”: a qualitative analysis of how yoga affects relationships. J Holist Nurs. 2014;32(2):67‐77. 24166108 10.1177/0898010113508466 PMC4196270

[42] Thompson IA , Wolf CP , Mott E , et al. Luna yoga: a wellness program for female counselors and counselors‐in‐training to foster self‐awareness and connection. J Creat Ment Health. 2018;13(2):169‐184.

[43] Cramer H , Ward L , Saper R , Fishbein D , Dobos G , Lauche R . The safety of yoga: a systematic review and meta‐analysis of randomized controlled trials. Am J Epidemiol. 2015;182(4):281‐293. 26116216 10.1093/aje/kwv071

[44] Awan RMDM , Laskowski ERMD . Yoga: safe for all? Mayo Clin Proc. 2019;94(3):385‐387. 30792065 10.1016/j.mayocp.2019.01.015

[45] Cramer H , Quinker D , Pilkington K , Mason H , Adams J , Dobos G . Associations of yoga practice, health status, and health behavior among yoga practitioners in Germany—results of a national cross‐sectional survey. Complement Ther Med. 2019;42:19‐26. 30670242 10.1016/j.ctim.2018.10.026

[46] Fishman L , Saltonstall E , Genis S . Understanding and preventing yoga injuries. Int J Yoga Ther. 2009;19:47‐53.

[47] Swain TA , McGwin G . Yoga‐related injuries in the United States from 2001 to 2014. Orthop J Sports Med. 2016;4(11):2325967116671703. 27896293 10.1177/2325967116671703 PMC5117171

[48] Cramer H . Yoga therapy in the German healthcare system. Int J Yoga Ther. 2018;28(1):133‐135. 10.17761/2018-00006 29741426

[49] Jain R . Certifications, registrations and insurance: what are the legal requirements for teaching yoga in your country and worldwide? 2019. Accessed January 1, 2023. https://www.arhantayoga.org/blog/required-certifications-insurance-teaching-yoga/

[50] Stussman BJ , Nahin RR , Barnes PM , Ward BW . U.S. physician recommendations to their patients about the use of complementary health approaches. J Altern Complement Med. 2020;26(1):25‐33. 31763927 10.1089/acm.2019.0303 PMC6998052

[51] Cramer H , Lauche R , Dobos G . Characteristics of randomized controlled trials of yoga: a bibliometric analysis. BMC Complement Altern Med. 2014;14:328. 25183419 10.1186/1472-6882-14-328 PMC4161862

[52] Lewith GT , Hyland M , Gray SF . Attitudes to and use of complementary medicine among physicians in the United Kingdom. Complement Ther Med. 2001;9(3):167‐172. 11926430 10.1054/ctim.2001.0475

[53] Cartwright T , Richards R , Edwards A , Cheshire A . Yoga4Health on Social Prescription: A Mixed Methods Evaluation. University of Westminster; 2019.

